# An Evaluation of Dentin’s Effect on the Antifungal Activity of MTA Cements

**Published:** 2007-04-01

**Authors:** Zahed Mohammadi, Abbasali Khademi, Mohammad Hossein Toodehzaeim

**Affiliations:** 1 *Department of Endodontics, Dental School, Shahid Sadoughi University of Medical Sciences, Yazd, Iran*; 2 *Department of Endodontics, Dental School, Isfahan University of Medical Sciences, Isfahan, Iran*; 3 *Department of Orthodontics, Dental School, Shahid Sadoughi University of Medical Sciences, Yazd, Iran*

**Keywords:** Candida Albicans, Dentin, Inactivation, MTA

## Abstract

**INTRODUCTION:** The aim of this study was to evaluate the effect of dentin on the antifungal activity of gray and white-colored MTA (GMTA, WMTA) using a tube-dilution test.

**MATERIALS AND METHODS:** MTA preparations were tested freshly mixed and after 24h on *Candida Albicans* (CA). The experiment was performed in 24-well culture plates. Fifty wells were used and divided into four experimental groups (freshly-mixed WMTA, freshly-mixed WMTA plus dentin powder, freshly-mixed GMTA, and freshly-mixed GMTA plus dentin powder) of 10 wells each and control groups of five wells each. Plates of Sabouraud dextrose agar mixed with CA served as positive control and Sabouraud dextrose agar without CA served as negative control. Fresh inoculate of CA was prepared by growing an overnight culture from a stock culture. Aliquots of CA were then taken from the stock culture and plated on the agar compound of the experimental and positive control group. All plates were incubated at 37°C for1h, 24h, and 72h. Growth of fungi was monitored daily by the presence of turbidity. Kruskal-Wallis test was used for statistical analysis.

**RESULTS:** Results showed the inhibitory effect of dentin powder on the antifungal effect of MTA cements during 24h and 72h incubation periods, whereas, there was no significant difference between 1h incubation groups.

**CONCLUSION:** The antifungal effect of freshly mixed MTA cements was decreased in the presence of dentin.

## INTRODUCTION

Microorganisms play an essential role in pulpal and periapical diseases (1-3). Throughout the past decades, it has been well known that yeasts can be isolated from infected root canals. The occurrence of yeasts reported in infected root canals varies between 1 and 17 percent (4).

It has been shown that fungi colonization resulting in radicular pathosis can be associated with failing root canal treatments (5-9). The most commonly recovered fungi were CA (5,9,10). CA also showed the ability to colonize root canal walls and penetrate into dentinal tubules.

Factors affecting the colonization of the root canal by fungi are not fully understood. However, it seems that among the predisposing factors of this process there are certain intracanal medicaments, local and systemic antibiotics, and previous unsuccessful RCT. It has been hypothesized that the reduction of specific bacteria in the root canal during endodontic treatment may allow fungi overgrowth in the low nutrition environment. Further, fungi, such as CA may gain access to the root canal because of coronal leakage (4).

Failures of initial endodontic treatment can often be successfully treated by orthograde retreatment or endodontic surgery. Elimination of the microbial flora and infected tissue as well as complete seal of the root canal system, in order to prevent future recontamination, will enhance treatment success (11). MTA has become a popular material to seal off communications between the root canal system and external surface of the root. It has been mostly used as a retrograde filling material and as a sealant of root perforations (12). Pro Root MTA is marketed in gray-coloured and white-coloured preparations; both are 75% Portland cement, 20% bismuth oxide, and 5% gypsum by weight (11). In recent years, the use of the white-coloured preparation became more popular. MTA is a powder that consists of fine hydrophilic particles that in the presence of water or moisture forms a colloidal gel that solidifies to form hard cement within approximately 3h (12). The main components of the gray-coloured formula are tricalcium oxide, tricalcium silicate, bismuth oxide, dicalcium, silicate, tricalcium aluminate, tetracalcium aluminoferrite, and calcium sulfate dihydrate. The white-colored preparation, however, lacks tetracalcium aluminoferrite (11). A number of studies have revealed the inhibitory effect dentin, dentin matrix and hydroxyapatite on the antimicrobial activity of several intracanal medicaments (i.e. calcium hydroxide). Considering the fact that calcium hydroxide is the main chemical compound produced by MTA cements in aqueous environments, evaluation of inhibitory effect of dentin on the antimicrobial effects of these cements seems to be interesting. The purpose of this *in vitro* study was to assess the inhibitory effect of dentin on the antifungal effects of MTA cements against *Candida Albicans*.

## MATERIALS AND METHODS

The antifungal activity of white as well as gray-coloured MTA (Pro Root MTA, Dentsply, Tulsa, OK, USA) was evaluated against *Candida Albicans (CA)*. Stock cultures of clinically isolated CA provided by the Microbiology Laboratory of Sadoughi Medical University (Yazd, Iran) were maintained in Sabouraud agar plate. A suspension was prepared by transferring three colonies from the Sabouraud agar plate using a sterile 4-mm diameter platinum loop to 10 mm of Sabouraud infusion broth in a sterilized 10 mm screw-capped test tube and then incubated at 37°C for 1 week. Dentin powder was prepared from extracted maxillary incisor teeth. After extraction, teeth were kept in 0.5% NaOCl for 24h to remove remnants of soft tissue. Before further preparation the teeth were rinsed and autoclaved. The crowns of the teeth were removed with a diamond saw under strict antiseptic conditions. Root canals of teeth were instrumented using sterile Haedsreoem files to produce dentin powder. The experiment was performed in plastic tissue-culture clusters containing 24 wells each with an inner diameter of 16 mm. A total of fifty wells were used and divided into four experimental groups (freshly-mixed WMTA, freshly-mixed GMTA, freshly-mixed WMTA plus dentin powder, and freshly-mixed GMTA plus dentin powder) of 10 wells each and control groups of five wells each. In the experimental group 1, 1 g of WMTA was mixed at the bottom of each culture well. In the experimental group 2, 1 g of GMTA was mixed at the bottom of each culture well. In groups 3 and 4, freshly mixed WMTA plus dentin powder, and freshly mixed GMTA plus dentin powder were used respectively. Fifty milligrams sterile dentin powder was added to the wells in groups 3 and 4 and was thoroughly mixed with a sterile pipette before adding the fungal inuculum. For the positive control group, 1mL of Sabouraud infusion-broth media (SIB) was mixed with 1mL of CA suspension in a culture well. In the negative control group, 2mL of SIB was placed in culture well. The culture plates of all experimental and control groups were then incubated anaerobically at 37°C and evaluated at 1, 24 and 72h time periods. At each time period, aliquots of 0.1mL were taken from each well and transferred to tubes containing 5mL of fresh SIB. All tubes were incubated at 37°C and monitored for the consecutive 7 days. Growth of the fungi was monitored daily by the presence of turbidity in the tubes. The results were analyzed statistically using Kruskal-Wallis test.

## RESULTS

The negative control showed no fungal growth in all experimental periods, whereas the positive control demonstrated entirely fungal growth, which confirms the method.

Evaluation of the freshly mixed MTA groups without dentin powder demonstrated fungal growth during the 1h incubation of CA with WMTA as well as GMTA. However, by increasing the incubation time, there was no growth in 24h and 72h. In the freshly mixed MTA groups containing dentin powder fungal growth was observed during all incubation periods. There was no statistical significant difference between GMTA and WMTA (P>0.05). However, the difference between groups containing dentin powder and groups without dentin powder was statistically significant (P<0.05). All cell culture wells had the same results in each experimental group.

## DISCUSSION

The method used in the present study is the dilution-tube-susceptibility test, which is an effective method to evaluate the antifungal properties of any filling material or solution (13). This method provides direct contact between fungal cells and the MTA material. Sabouraud agar is a commonly used medium for the isolation of oral yeasts. The pH of the medium is quite acidic (usually 5.6) allowing the growth of yeasts and acid uric organisms, whereas most bacteria are inhibited (4).

CA frequently associated with failing root canal treatments, has the ability to form biofilm on different surfaces (5). This property is one of the reasons why this species is considered to be more pathogenic than species that are less able to form biofilm (5).

There is no published study regarding the inhibitory effects on dentin or its constituents on the antimicrobial activity of MTA cements.

However, a number of studies have evaluated the buffering effect of dentin and its elements on the antimicrobial activity of intracanal medications and root canal irrigants. Haapasalo *et al.* (14) evaluated the effect of dentin powder on the antibacterial activity of saturated calcium hydroxide solution, 1% sodium hypochlorite, 0.05% and 0.5% chlorhexidine, and 0.2/0.4% iodine potassium iodine. Results showed that dentin powder had an inhibitory effect on all medicaments tested. The effect was dependent on the concentration of the medicament as well as the length of time the medicament was pre-incubated with dentin powder before adding the bacteria. In another study, Portenier *et al.* (15) evaluated the effect of dentin, dentin matrix, type I collagen, and heat-killed microbial whole cells on the antibacterial activity of chlorhexidine diglucanate and iodine potassium iodide. Findings showed that dentin matrix and heat-killed microbial cells were the most effective inhibitors of chlorhexidine. Dentin and collagen showed some inhibition at 1^st^ hour but not after 24h. Iodine potassium iodide was effectively inhibited by dentin, dentin matrix, and heat-killed microbial cells.

The results of present study showed that freshly mixed and 24h set MTA cements without dentin powder were effective against CA in 24 and 72h time periods. Al-Nazhan and Al-Judai (14) showed that white-coloured MTA to be effective in killing CA *in vitro* for a period of up to 72h. On the other hand, Estrella *et al*. (15) found that the antifungal activity of gray-coloured MTA against CA was limited during a 48h period.

In another in vitro study, Sipert *et al.* (16) found that antifungal effect of MTA was not significantly different from Portland cement, but was significantly less than root canal sealers. To date, there is no evidence of the antifungal activity of MTA against *CA* for periods longer than 3 days. However, it has been shown that MTA has a soluble fraction mainly composed of calcium hydroxide and the water in contact with MTA had a high alkaline PH ranging from 11.94 to 11.99 (17). A long-term study demonstrated that MTA did maintain a high pH for 78 days (18). It is therefore possible that MTA and calcium hydroxide possess similar antimicrobial action. In this regards, several studies evaluated the susceptibility of CA to calcium hydroxide. Waltimo *et al*. (19) studied the susceptibility of common oral *Candida *species to saturated aqueous calcium hydroxide solution. They found that the sensitivity of the CA strains was relatively low for short-term exposure; however, after 6h of incubation, 99.9% of *Candida* strains were killed. On the other hand, Barbosa *et al*. (20) found that a saturated solution of calcium hydroxide was effective in killing CA already after 3min of incubation. Siqueira *et al*. (21) evaluated the antifungal activity of different intracanal medicaments against CA and found that calcium hydroxide completely eliminated the fungus from bovine dentin after 7 days. They also found that a combination of calcium hydroxide and paramonochlorophenol eliminated CA within 1h. Estrella *et al.* (15) reported that an aqueous preparation of calcium hydroxide paste in saline was more effective in inhibiting CA growth than MTA or Portland cement. Al-Hezaimi *et* *al.* (11) evaluated the antifungal activity of different concentrations of white-coloured MTA on CA *in vitro *in various exposure periods. Their findings showed a direct correlation between MTA concentration and its inhibition effect on the growth of CA. MTA in concentration of 50mg/mL inhibited the growth of CA in any of the time periods tested (1,24,48,72h). In concentration of 25mg/ml, MTA inhibited the growth of CA at 1 and 24-h time periods. In lower concentrations, MTA did not inhibit the growth of CA.

Extrapolation of the results of this in vitro study to clinical situations must be done with caution. Sealing ability and biocompatibility of MTA are more important in clinical practice. Further, the 72h evaluation of the MTA cements is not sufficient for any conclusions to be drawn about their antifungal effects. Therefore, it is suggested that the antifungal activity of MTA cements be investigated for longer periods of time.

## CONCLUSION

Within the limits of the present in vitro study, antifungal activity of white-colored as well as gray-colored MTA was completely inhibited in the presence of dentin powder.
